# Higher mortality associated with the SARS-CoV-2 Delta variant in the Western Cape, South Africa, using RdRp target delay as a proxy: a cross-sectional study.

**DOI:** 10.12688/gatesopenres.13654.1

**Published:** 2022-08-31

**Authors:** Hannah Hussey, Mary-Ann Davies, Alexa Heekes, Carolyn Williamson, Ziyaad Valley-Omar, Diana Hardie, Stephen Korsman, Deelan Doolabh, Wofgang Preiser, Tongai Maponga, Arash Iranzadeh, Susan Engelbrecht, Sean Wasserman, Neshaad Schrueder, Linda Boloko, Greg Symons, Peter Raubenheimer, Abraham Viljoen, Arifa Parker, Cheryl Cohen, Waasila Jasat, Richard Lessells, Robert J Wilkinson, Andrew Boulle, Marvin Hsiao

**Affiliations:** 1 Health Intelligence, Western Cape Government: Health, Cape Town, South Africa; 2 Division of Public Health Medicine, School of Public Health and Family Medicine, University of Cape Town, Cape Town, South Africa; 3 Centre for Infectious Disease Epidemiology and Research, School of Public Health and Family Medicine, University of Cape Town, Cape Town, South Africa; 4 Division of Medical Virology, University of Cape Town, Cape Town, South Africa; 5 Wellcome Centre for Infectious Diseases Research in Africa, Institute of Infectious Disease and Molecular Medicine, University of Cape Town, Cape Town, South Africa; 6 National Health Laboratory Service, Cape Town, South Africa; 7 Division of Medical Virology, Stellenbosch University, Cape Town, South Africa; 8 Division of Infectious Diseases and HIV Medicine, Department of Medicine, University of Cape Town, Cape Town, South Africa; 9 Department of Medicine, Tygerberg Hospital,, Stellenbosch University, Cape Town, South Africa; 10 Department of Medicine, Groote Schuur Hospital, University of Cape Town, Cape Town, South Africa; 11 National Institute for Communicable Diseases, Johannesburg, South Africa; 12 School of Public Health, Faculty of Health Sciences, University of the Witwatersrand, Johannesburg, South Africa; 13 KwaZulu-Natal Research, Innovation & Sequencing Platform, University of KwaZulu Natal, Durban, South Africa; 14 The Francis Crick Institute, London, UK; 15 Department of Infectious Diseases, Imperial College London, London, UK

**Keywords:** SARS-CoV-2, Delta, B.1.617.2, clinical severity, RdRp target delay, South Africa

## Abstract

**Background:** The SARS-CoV-2 Delta variant (B.1.617.2) has been associated with more severe disease, particularly when compared to the Alpha variant. Most of this data, however, is from high income countries and less is understood about the variant’s disease severity in other settings, particularly in an African context, and when compared to the Beta variant.

**Methods:** A novel proxy marker, RNA-dependent RNA polymerase (RdRp) target delay in the Seegene Allplex ^TM^ 2019-nCoV (polymerase chain reaction) PCR assay, was used to identify suspected Delta variant infection in routine laboratory data. All cases diagnosed on this assay in the public sector in the Western Cape, South Africa, from 1 April to 31 July 2021, were included in the dataset provided by the Western Cape Provincial Health Data Centre (PHDC). The PHDC collates information on all COVID-19 related laboratory tests, hospital admissions and deaths for the province. Odds ratios for the association between the proxy marker and death were calculated, adjusted for prior diagnosed infection and vaccination status.

**Results:** A total of 11,355 cases with 700 deaths were included in this study. RdRp target delay (suspected Delta variant) was associated with higher mortality (adjusted odds ratio [aOR] 1.45; 95% confidence interval [CI]: 1.13-1.86), compared to presumptive Beta infection. Prior diagnosed infection during the previous COVID-19 wave, which was driven by the Beta variant, was protective (aOR 0.32; 95%CI: 0.11-0.92) as was vaccination (aOR [95%CI] 0.15 [0.03-0.62] for complete vaccination [≥28 days post a single dose of Ad26.COV2.S or ≥14 days post second BNT162b2 dose]).

**Conclusion:** RdRp target delay, a proxy for infection with the Delta variant, is associated with an increased risk of mortality amongst those who were tested for COVID-19 in our setting.

## Introduction

To date, the Western Cape Province of South Africa, has experienced four large waves of SARS-CoV-2 infections. The first wave caused by early ancestral clades of SARS-CoV-2 peaked in June 2020, the second wave with the Beta variant (B.1.351) peaked in December 2020, the third wave caused by the Delta variant (B.1.617.2) peaked in August 2021 and the fourth most recent wave was caused by the Omicron variant peaked in December 2021 ^1^.

Prior to the arrival of Omicron, Delta had been the dominant variant globally, its increased clinical severity particularly when compared to Alpha is well documented ^2– 6^. Most of the severe disease in these studies occurred in those who were unvaccinated. The concern is that in these settings with well-established vaccination programmes, there are differences between the vaccinated and unvaccinated population, largely driven by socioeconomic disparities ^7^, that might contribute to the clinical severity seen and confound the association between variant and disease severity. In addition, there is only limited data on Delta disease severity within a sub-Saharan African setting.

The start of the third wave of COVID-19 in the Western Cape Province was characterized by a rapid transition from the previously dominant Beta variant to Delta ^8^. Using a novel proxy marker for Delta, namely RNA-dependent RNA polymerase (RdRp) target delay (RTD) in (polymerase chain reaction) PCR positive samples, our objective was to assess mortality associated with Delta, compared to Beta, in our population which had relatively low levels of vaccine coverage as well as a high prevalence of comorbidities, including HIV and tuberculosis.

## Methods

### RdRP target delay

RTD, defined as a difference in cycle threshold (Ct) value of >3.5 in the RdRp relative to E gene target in the Seegene Allplex ^TM^ 2019-nCoV PCR assay (Seegene Inc, USA), has recently been described ^9^. This phenomenon is due to the G15451A mutation found in Delta, resulting in a RdRp primer mismatch ^9^. When evaluated against genomic data, this method had a sensitivity of 93.6% and specificity of 89.7% in detecting Delta ^9^. RTD has been used to assess variant disease severity in our setting during the fourth wave ^10^.

### Population and statistical analysis

We included all COVID-19 cases diagnosed on the Seegene Allplex ^TM^ 2019-nCoV diagnostic PCR assay in the Western Cape public sector from 1 April to 31 July 2021, a period when both Beta and Delta were co-circulating ( Appendix Figure 1) All available cases were included without sampling. Alpha and non-variant of concern lineages accounted for a negligible number of infections at this time ^8^. Follow-up ended on 31 August 2021, at which point most of the expected COVID-19 related outcomes would have occurred. Approximately 70% of the Western Cape population uses the public sector for health services and the Western Cape Provincial Health Data Centre (PHDC) collates all available electronic health data on these patients ^11^. The PHDC combines laboratory data on COVID-19 tests with hospital admission and death data, as well as information on known comorbidities and where available, vaccination status. Use of de-identified linked data provided by the PHDC in COVID-19 analyses has been previously described ^12^. COVID-19 deaths were defined analytically as deaths within 28 days of diagnosis of COVID-19 or 14 days after discharge from hospital (where hospital admission occurred within 21 days of the COVID-19 diagnosis), without a non-COVID-19 cause of death recorded by the health facility or COVID-19 case managers. Out-of-facility deaths were determined by civil identifier linkage of patients with COVID-19 diagnoses to the national population register.

We undertook logistic regression using Stata 13.1 (Stata corp, 2013) (RRID:SCR_012763) to determine the association between RTD and mortality, adjusted for age, sex, known comorbidities, prior diagnosed infection, vaccination status at time of diagnosis, sub-district of residence, month of diagnosis and hospital admission pressure (number of public sector admissions in the calendar week of diagnosis, categorized into four [weekly admission of ≤350, ≤700, ≤1000 and >1000]). All variables were included as binary or categorical variables. Prior diagnosed infections were defined as a positive COVID-19 test more than 90 days prior to the current test and classified into those with their first infection in the first wave (1 March – 30 September 2020) or second wave (1 October 2020 – 31 March 2021). Vaccination status at time of COVID-19 diagnosis was determined by the PHDC prior to de-identification by linking COVID-19 cases with the national Electronic Vaccination Data System (EVDS) through national civil identifiers in both the PHDC and EVDS databases. EVDS, a national clinical data system that is not publicly accessible, is one of the data sources integrated into the PHDC ^11^. For the purposes of this study, we defined complete vaccination analytically as ≥28 days post-vaccination with Janssen/Johnson & Johnson (Ad26.COV2.S), or ≥14 days post second dose of Pfizer–BioNTech (BNT162b2). Patients were deemed partially vaccinated from the day after their (first) vaccine dose until meeting criteria for complete vaccination.

A secondary analysis was done, using the same methodology as above, but stratifying the cases into those aged less than 50 years and those aged 50 years and above.

## Ethical approval

The study was approved by the University of Cape Town Research Ethics Committee (HREC 460/2020).

## Results

We included 11,355 cases tested using the Seegene Allplex ^TM^ assay, which were 22% of all positive test results in the province in that time period ( Appendix Table 1). The median age was 43 years (interquartile range [IQR] 32-55) and 44% were male. RTD was present in 9106 (80%) of the cases included ( Table 1). Patient characteristics were similar in those with and without RTD. There was, however, a difference in Ct values (average of the E and N gene targets); those with RTD had lower median Ct values (26.1, IQR 22.2-30.9, vs 32.7 IQR 25.6-37.6).

**Table 1.** Characteristics of all COVID-19 cases (Seegene Allplex ^TM^ 2019-nCoV assay positive) in the Western Cape, April – July 2021, by presence of RNA-dependent RNA polymerase (RdRP) target delay (RTD).

		All cases (n=11,355; 700 deaths)		RdRP Target Delay (n=9,106; 570 deaths)		No RdRP Target (n=2,249; 130 deaths)	
		n	%	n	%	n	%
**RTD**	**Absent**	2249	19.8				
	**Present**	9106	80.2				
**Sex**	**Female**	6411	56.5	5143	56.5	1268	56.4
	**Male**	4944	43.5	3963	43.5	981	43.6
**Age category**	**20–29 years**	2243	19.8	1810	19.9	433	19.3
	**30–39 years**	2623	23.1	2149	23.6	474	21.1
	**40–49 years**	2427	21.4	1956	21.5	471	20.9
	**50–59 years**	2113	18.6	1701	18.7	412	18.3
	**60–69 years**	1177	10.4	899	9.9	278	12.4
	**≥70 years**	772	6.8	591	6.5	181	8.1
**Comorbidities**	**HIV positive**	726	6.4	560	6.2	166	7.4
	**Diabetes**	1546	13.6	1218	13.4	328	14.6
	**Current tuberculosis**	94	0.8	66	0.7	28	1.2
	**Hypertension**	2475	21.8	1963	21.6	512	22.8
	**Chronic Kidney Disease**	374	3.3	299	3.3	75	3.3
	**Chronic Obstructive Pulmonary** ** Disease**	876	7.7	685	7.5	191	8.5
**Prior diagnosed** ** infection**	**None**	11107	97.8	8914	97.9	2193	97.5
	**First infection in first wave**	64	0.6	46	0.5	18	0.8
	**Second infection in second wave**	184	1.6	146	1.6	38	1.7
**Vaccination status at** ** time of diagnosis ¶**	**Unvaccinated**	10422	91.8	8358	91.8	2064	91.8
	**Partially vaccinated**	694	6.1	544	6.0	150	6.7
	**Completely vaccinated**	239	2.1	204	2.2	35	1.6

¶ Fully vaccinated was defined as ≥28 days post-vaccination with Janssen/Johnson & Johnson or ≥14 days post second dose of Pfizer–BioNTech. Patients were deemed partially vaccinated from the day after their (first) vaccine dose until meeting criteria for complete vaccination.

Amongst the Seegene Allplex ^TM^ cases in our study, there were 700 deaths meeting the above definition for COVID-19-associatedness, with a case fatality rate of 6.3% in those with RTD, compared to 5.8% in those without. After adjusting for all covariates, we found that RTD was associated with death among cases (aOR 1.45 [95% CI 1.13-1.86]) and among those admitted to hospital (aOR 1.39 [95% CI 1.03-1.88]) ( Table 2). 

Prior diagnosed infection with a first infection in the Beta dominated second wave (vs no prior diagnosed infection), was protective against death (aOR 0.32 [95% CI 0.11-0.92]), whereas prior diagnosed infection with a first infection in the first wave was not (aOR: 1.56 [95% CI 0.50-4.92]). Vaccination was protective against death. The aORs (95% CI) for partial and full vaccination were 0.59 (0.45-0.77) and 0.15 (0.03-0.62) respectively.

**Table 2.** Logistic regression for outcome of death in a) all positive Seegene Allplex ^TM^ cases; b) restricted to those admitted to hospital only; c) stratified by age into those aged under 50 and over 50 years.

		Adjusted ^§^ OR (95%CI) for outcome of death			
		All cases (n=11,355; 700 deaths)	Only admitted cases * (n=1856; 612 deaths)	All cases < 50 years (n=7293; 113 deaths)	All cases ≥ 50 years (n=4062; 587 deaths)
**RTD**	**absent**	**Ref**	**Ref**	**Ref**	**Ref**
	**present**	**1.45 (1.13-1.86)**	**1.39 (1.03-1.88)**	**1.32 (0.77-2.26)**	**1.44 (1.09-1.91)**
**Sex**	**female**	**Ref**	**Ref**	**Ref**	**Ref**
	**male**	**1.50 (1.25-1.80)**	**1.19 (0.95-1.47)**	**1.16 (0.78-1.72)**	**1.63 (1.33-2.00)**
**Age category**	**20–29 years**	**Ref**	**Ref**	**Ref**	
	**30–39 years**	**2.75 (1.19-6.31)**	**2.19 (0.81-5.93)**	**2.49 (1.07-5.78)**	
	**40–49 years**	**6.89 (3.15-15.05)**	**5.91 (2.27-15.54)**	**6.48 (2.92-14.37)**	
	**50–59 years**	**11.49 (5.31 - 24.88)**	**6.86 (2.66-17.67)**		**Ref**
	**60–69 years**	**24.64 (11.36-53.42)**	**11.75 (4.56-30.28)**		**2.16 (1.68-2.79)**
	**≥70 years**	**72.81 (33.54-158.06)**	**25.93 (10.03-67.01)**		**6.43 (4.96-8.33)**
**Comorbidities †**	**HIV positive**	**1.66 (1.11-2.47)**	**1.14 (0.69-1.88)**	**2.21 (1.27-3.85)**	**0.99 (0.53-1.85)**
	**Diabetes**	**2.60 (2.14-3.16)**	**1.19 (0.95-1.49)**	**2.58 (1.55-4.32)**	**2.61 (2.11-3.22)**
	**Current tuberculosis**	**3.62 (1.76-7.42)**	**1.57 (0.68-3.61)**	**4.90 (1.90-12.65)**	**2.38 (0.78-7.20)**
	**Hypertension**	**1.13 (0.93-1.38)**	**0.95 (0.76-1.20)**	**1.07 (0.63-1.82)**	**1.16 (0.93-1.43)**
	**Chronic Kidney Disease**	**2.17 (1.65-2.86)**	**1.76 (1.29-2.42)**	**9.33 (4.20-20.72)**	**1.87 (1.41-2.50)**
	**Chronic Obstructive ** **Pulmonary Disease**	**1.27 (1.00-1.62)**	**0.91 (0.69-1.19)**	**1.95 (1.08-3.53)**	**1.17 (0.90-1.52)**
**Prior diagnosed** ** infection**	**None**	**Ref**	**Ref**	**Ref**	**Ref**
	**First infection in first ** **wave**	**1.56 (0.50-4.92)**	**2.63 (0.51-13.64)**	**1.11 (0.14-78.87)**	**2.12 (0.49-9.17)**
	**First infection in** ** second wave**	**0.32 (0.11-0.92)**	**0.34 (0.10-1.09)**	**0.68 (0.15-3.19)**	**0.14 (0.03-0.67)**
**Vaccination status at ** **time of diagnosis ¶**	**Unvaccinated**	**Ref**	**Ref**	**Ref**	**Ref**
	**Partially vaccinated**	**0.59 (0.45-0.77)**	**0.79 (0.57-1.09)**	**1.47 (0.32-6.66)**	**0.57 (0.44-0.75)**
	**Completely vaccinated**	**0.15 (0.03-0.62)**	**0.14 (0.02-1.20)**	**(omitted)**	**0.22 (0.05-0.96)**

§ Adjusted for all variables in the model, as well as month of diagnosis, sub-district of residence and number of COVID-19 admissions in week of diagnosis. * Date of hospital admission within 21 days of COVID-19 date of diagnosis. † The reference group here is the absence of that specific comorbidity. ¶ Fully vaccinated was defined as ≥28 days post-vaccination with Janssen/Johnson & Johnson or ≥14 days post second dose of Pfizer–BioNTech. Patients were deemed partially vaccinated from the day after their (first) vaccine dose until meeting criteria for complete vaccination.

In a stratified analysis the association between RTD and death was similar in both those aged <50 and ≥50 years (aOR [95%CI] of 1.32 [0.77-2.26] and 1.44 [1.09-1.91] respectively).

## Discussion

RTD, a proxy marker for the Delta variant, was associated with an increased risk for death compared to presumptive Beta variant infection, while prior diagnosed infection (with first infection in the second wave) and vaccination were strongly protective. These findings are corroborated by a recent study from the Western Cape, which is the only other study to assess mortality with the Delta variant to compared to Beta. In that analysis, the wave itself was used as a proxy for variant, and the third wave (“Delta”: 26 May - 23 June 2021) had a higher aOR for mortality when compared to the second wave (“Beta”: 25 October - 21 November 2020) of 1.79 (95%CI 1.41-2.27) ^13^, which is slightly higher than this analysis. Although the confidence intervals do overlap, the possible difference might be explained by the inclusion of cases after testing restrictions were in place in this analysis.

The increased transmissibility of Delta is well established, and may be due to its higher viral load ^6^. In our analysis, specimens with RTD had lower Ct values than those without, suggesting a higher viral load. This, along with immune evasion by Delta, could contribute to the increased disease severity seen ^14^. Nonetheless, our results need to be interpreted in the context of our setting where not all COVID-19 cases would have accessed testing. Delta often results in mild symptoms and during wave surges, as was the case from 15 June 2021 until the end of the analysis period, in the public sector only those aged ≥45 years or with comorbidities or requiring hospital admission would be eligible for all SARS-CoV-2 testing ^15^. The interpretation of our results is therefore, that among those who access a PCR test, the Delta variant is associated with worse outcomes. While it is unclear if this finding can be extrapolated to all those with COVID-19 in our setting, similar findings from countries with more widespread testing support the increased clinical severity of Delta ^2– 6^.

Hospital admission was more likely with Delta compared to Alpha in England (aHR 2.26 [95%CI 1.32-3.89]) ^2^ and Denmark (RR 2.83 [95%CI 2.02-3.98]) ^3^. In Singapore, infection with Delta and Beta vs. wild-type SARS-CoV-2 were both associated with a composite outcome of oxygen use, intensive care admission and death (aOR 4.9 [95%CI 1.43-30.78] and 1.69 [95%CI 0.19-14.69] respectively) ^4^. Similarly, a recent study from Qatar found that Delta compared to Beta had an aOR of 3.61 (1.65-7.91) for severe-critical disease, which was defined as intensive care unit admission, use of high-flow oxygen, mechanical ventilation, or death ^16^. Delta (vs wild-type) was associated with mortality in Canada (aOR 2.32 [95% CI 1.47-3.30]) as were N501Y-positive variants (Alpha, Beta, Gamma) (aOR 1.51 [95%CI 1.30-1.74]) ^5^.

However, most of these countries had relatively high COVID-19 vaccination coverage rates by the time the Delta variant became dominant there ^17^. Since most severe COVID-19 cases would be in unvaccinated people, differences between the vaccinated and the unvaccinated population might confound associations with variant infection and disease severity. By contrast in the Western Cape by 31 July 2021, only 5.0% of all adults, mostly in older age groups, were fully vaccinated ^17^. In addition, most of these studies compared the outcomes of Delta to Alpha. The Alpha variant has been shown to have worse clinical outcomes compared to wild-type, with an aHR for death of 1.73 (95%CI 1.41 - 2.13) ^18^.

Despite Delta no longer being the dominant variant, it continues to circulate globally and understanding and quantifying the disease severity associated with it and its spectrum of mutations remains critical. A Delta resurgence cannot be excluded, particularly with the recent emergence of the Omicron-Delta recombinant strain (BA.1 x AY.4) that the World Health Organisation has declared to be a variant under monitoring ^19^. In addition, disease severity of dominant circulating variants is often calculated relative to the previously dominant variants ^20^. The Omicron variant, for instance, has been found to cause less disease than the Delta variant, but this has to be understood in the context of the Delta variant already being associated with more severe disease compared to Alpha or Beta, hence quantifying the clinical severity of different variants is important for such comparisons ^10, 13^.

This study has several limitations. First, we could only include cases tested on the Seegene Allplex ^TM^ 2019-nCoV assay, excluding cases diagnosed by other PCR methods or antigen testing. However, the included cases are mostly representative of all diagnosed PCR cases in the Western Cape ( Appendix Table 1). Patients tested on this assay were similar in age, sex, known comorbidities, prior diagnosed infection and vaccination status to those who tested positive using other PCR assays. As different laboratories service different geographical regions there was a difference, however, in the district of residence. Patients who tested positive on antigen tests tended to be younger, have fewer comorbidities and fewer were admitted to hospital. This is in accordance with the Western Cape’s testing guidelines where PCR was preferred for hospital patients, while antigen testing was promoted at primary health care facilities ^15^.

Second, while RTD is a reliable proxy marker for Delta, it is not as accurate as whole genome sequencing, and misclassification may have diluted the effect of Delta. Third, we could only assess the effect of prior laboratory-confirmed COVID-19 infection and seroprevalence studies suggest considerably higher numbers were infected, even after the first wave, and that prior infection prevalence differed by sub-district of residence ^21^. While we did adjust for sub-district, the absence of a protective effect of prior infection in the first wave may be due to insufficient numbers of those infections being diagnosed. Fourth, the inclusion of cases after testing restrictions were introduced is likely to result in an underestimate of Delta disease severity. However, in the stratified analysis, those younger than 50 years, who would be more likely to be affected by the testing restrictions, still had a similar findings to those >50 years. Fifth, although we adjusted for COVID-19 hospital admissions to account for escalating service pressure during the wave surge, we could not adjust for non-COVID-19 admissions that might have added to pressure on facilities and contributed to mortality.

And finally, there are innate limitations in using observational data from surveillance and routine health records to assess variant disease severity, particularly as potential biases around testing patterns cannot always be fully adjusted for 20.

## Conclusion

In this study we found that RTD, a useful proxy for infection with the Delta variant, is associated with an increased risk of mortality amongst those who were tested for COVID-19 in our setting. This suggests that the Delta variant is associated with an increased risk of mortality when compared with other variants, and the Beta variant in particular.

## Data availability

The Western Cape Provincial Health Data Centre collates all available electronic health data on public sector patients in the province for operational service use. The underlying data in this study are routinely collected patient records that have been de-identified and pseudo-anonymised in accordance with research ethics requirements. The patients have not consented to these data being part of publicly accessible repositories considering the inherent risks of re-identification. The Western Cape Department of Health and Wellness evaluates research proposals for all research in the public health sector in the province, subject to standard research ethics, government approval and data governance prescripts. This includes those that draw on routine datasets like the current study. For more information on conducting research in health services or on data managed by the Western Cape Government Department of Health and Wellness (WCGHW), please email health.research@westerncape.gov.za. 

## Figures and Tables

**Figure 1.**
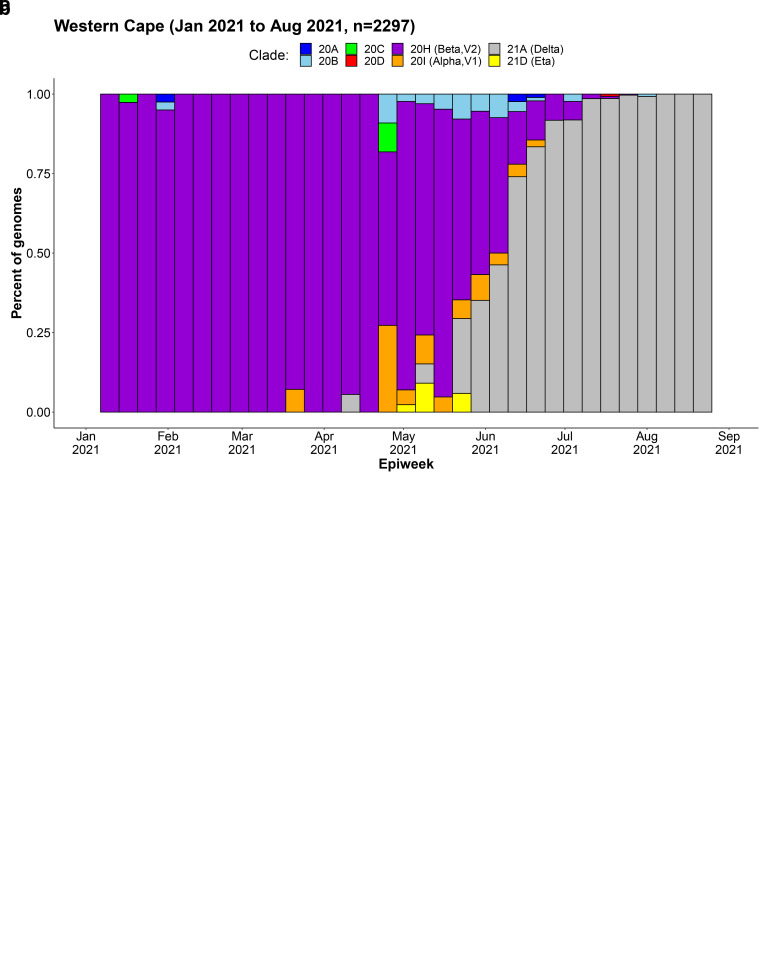
Weekly frequency and distribution of SARS-CoV-2 variants circulating in the Western Cape Province, South Africa, 1 January to 31 August 2021: **a**) absolute count of genomes sequenced, **b**) proportion of genomes sequenced ^9^.

**Table 1.** All positive SARS-CoV-2 tests in the Western Cape public sector, April to July 2021.

	Total positive tests (n=50,768)	Seegene AllplexTM PCR (n=11,355; 22.4%)		Other PCR tests (n=25,042; 49.3%)		Antigen tests (n=14,371; 28%)	
		n	%	n	%	n	%
**Month of diagnosis**	**April**	165	1.5	900	3.6	106	0.7
	**May**	302	2.7	1260	5.0	637	4.4
	**June**	2161	19.0	5223	20.9	2836	19.7
	**July**	8727	76.9	17659	70.5	10792	75.1
**District of residence**	**Cape Winelands**	508	4.5	2449	9.8	3125	21.8
	**Central Karoo**	246	2.2	431	1.7	302	2.1
	**City of Cape Town**	4975	43.8	12039	48.1	8954	62.3
	**Garden Route**	4642	40.9	4063	16.2	961	6.7
	**Overberg**	426	3.8	2448	9.8	522	3.6
	**West Coast**	557	4.9	3503	14.0	486	3.4
	**Unallocated**	1	0.0	109	0.4	21	0.2
**Sex**	**Male**	4944	43.5	10602	42.3	6427	44.7
**Age category**	**20–29 years**	2243	19.8	4805	19.2	2711	18.9
	**30–39 years**	2623	23.1	5497	22.0	3410	23.7
	**40–49 years**	2427	21.4	5200	20.8	3025	21.1
	**50-59 years**	2113	18.6	5104	20.4	3187	22.2
	**60–69 years**	1177	10.4	2660	10.6	1241	8.6
	**≥70 years**	772	6.8	1776	7.1	797	5.6
**Comorbidities**	**HIV positive**	726	6.4	1586	6.3	405	2.8
	**Diabetes**	1546	13.6	3433	13.7	1488	10.4
	**Current tuberculosis**	94	0.8	284	1.1	60	0.4
	**Hypertension**	2475	21.8	5357	21.4	2272	15.8
	**Chronic Kidney Disease**	374	3.3	702	2.8	270	1.9
	**Chronic Obstructive Pulmonary Disease**	876	7.7	2115	8.5	878	6.1
**Prior diagnosed infection**	**None**	11107	97.8	24339	97.2	14245	99.1
	**First infection in first wave**	64	0.6	202	0.8	43	0.3
	**First infection in second wave**	184	1.6	501	2.0	83	0.6
Vaccination status at time of diagnosis *	**Unvaccinated**	10422	91.8	22930	91.6	13223	92.0
	**Partially vaccinated**	694	6.1	1457	5.8	904	6.3
	**Completely vaccinated**	239	2.1	655	2.6	244	1.7
**Admitted ^§^**	**Case admitted to hospital**	1856	16.4	4964	19.8	1649	11.5

* Fully vaccinated was defined as ≥28 days post-vaccination with Janssen/Johnson & Johnson or ≥14 days post second dose of Pfizer–BioNTech. Patients were deemed partially vaccinated from the day after their (first) vaccine dose until meeting criteria for complete vaccination. § Admission with 21 days before or after COVID-19 diagnosis date
