# Supplementary material for: Higher mortality associated with the SARS-CoV-2 Delta variant in the Western Cape, South Africa, using RdRp target delay as a proxy: a cross-sectional study
Source: Gates Open Res. 2022 Aug 31;6:117. [Version 1] doi: 10.12688/gatesopenres.13654.1 (PMC10663174; doi:10.12688/gatesopenres.13654.1)
Supplement: Peer review history

# Reviewer report: Reviewer response for version 1

**DOI:** 10.21956/gatesopenres.14936.r35389

**Published:** 2023-11-21

**Authors:** Polani Rubeshkumar

**Affiliations:** 1 Public Health Wales NHS Trust, Cardiff, UK

This is a detailed and thorough investigation into the mortality rates associated with the Delta variant of SARS-CoV-2 in South Africa. The study is based on a cross-sectional analysis and utilizes the RdRp target delay in PCR testing as a proxy for identifying suspected Delta variant infections. It contributes significantly to the understanding of COVID-19 variant impacts in a lower-middle-income country setting. The study's methodology, data analysis, and contextual focus are commendable, although the limitations typical of observational studies should be considered when interpreting the findings.

 Minor comment:

 Just to ensure uniformity, I suggest the authors to use either mortality or deaths in the manuscript text.

Is the work clearly and accurately presented and does it cite the current literature?

Yes

If applicable, is the statistical analysis and its interpretation appropriate?

Yes

Are all the source data underlying the results available to ensure full reproducibility?

Yes

Is the study design appropriate and is the work technically sound?

Yes

Are the conclusions drawn adequately supported by the results?

Yes

Are sufficient details of methods and analysis provided to allow replication by others?

Yes

Reviewer Expertise:

Field Epidemiology; Infectious disease; Tropical medicine

I confirm that I have read this submission and believe that I have an appropriate level of expertise to confirm that it is of an acceptable scientific standard.

# Reviewer report: Reviewer response for version 1

**DOI:** 10.21956/gatesopenres.14936.r32523

**Published:** 2022-10-10

**Authors:** Jeannie Collins

**Affiliations:** 1 Medical Research Council Clinical Trials Unit, University College London, London, UK

This is an important paper on higher mortality associated with SARS CoV-2 Delta variant using data from the Western Cape provincial health data. The paper is well written, the conclusions are well supported and the potential limitations clearly stated. 

 In brief, the cross sectional study utilised a novel proxy marker for Delta variant (RdRP target delay) and assessed the risk of mortality and hospital admissions among cases with Delta variant versus those without (assumed to be Beta which was circulating at the time).  The analyses were adjusted for key characteristics including age, known comorbidities, prior SARS CoV-2 diagnosis and vaccination status. Stratified analyses by age group had broadly similar results.  

 Minor comment: 

 In the methods the authors could note how the RdRP target delay tests were distributed and used (versus other PCR tests), was this randomly allocated to patients or health care facilities? As there were some variations in the district of residence, as noted in the Discussion.

Is the work clearly and accurately presented and does it cite the current literature?

Yes

If applicable, is the statistical analysis and its interpretation appropriate?

Yes

Are all the source data underlying the results available to ensure full reproducibility?

No

Is the study design appropriate and is the work technically sound?

Yes

Are the conclusions drawn adequately supported by the results?

Yes

Are sufficient details of methods and analysis provided to allow replication by others?

Yes

Reviewer Expertise:

infectious disease epidemiology

I confirm that I have read this submission and believe that I have an appropriate level of expertise to confirm that it is of an acceptable scientific standard.
